# Change Detection & Characterization: a New Tool for Imaging Informatics and Cancer Research

**Published:** 2007-05-12

**Authors:** Julia W Patriarche, Bradley J Erickson

## Abstract

Modern imaging systems are able to produce a rich and diverse array of information, regarding various facets of anatomy and function. The quantity of information produced by these systems is so bountiful, however, as to have the potential to become a hindrance to clinical assessment. In the context of serial image evaluation, computer-based change detection and characterization is one important mechanism to process the information produced by imaging systems, so as to reduce the quantity of data, direct the attention of the physician to regions of the data which are the most informative for their purposes, and present the data in the form in which it will be the most useful. Change detection and characterization algorithms may serve as a basis for the creation of an objective definition of progression, which will reduce inter and intra-observer variability, and facilitate earlier detection of disease and recurrence, which in turn may lead to improved outcomes. Decreased observer variability combined with increased acuity should make it easier to discover promising therapies. Quantitative measures of the response to these therapies should provide a means to compare the effectiveness of treatments under investigation. Change detection may be applicable to a broad range of cancers, in essentially all anatomical regions. The source of information upon which change detection comparisons may be based is likewise broad. Validation of algorithms for the longitudinal assessment of cancer patients is expected to be challenging, though not insurmountable, as the many facets of the problem mean that validation will likely need to be approached from a variety of vantage points. Change detection and characterization is quickly becoming a very active field of investigation, and it is expected that this burgeoning field will help to facilitate cancer care both in the clinic and research.

## Introduction

Since the advent of cancer therapies, there has been a need to assess the outcome of interventions. These assessments may be viewed generally to serve two purposes—in the clinic, assessments are made on individuals so that doctors can manage care appropriately; in the context of research, assessments are made of cohorts of patients, to assess response to therapies. The goal is the same in both contexts: the status of the patient and the tumor are to be monitored, and typically this is accomplished by comparing some observation or measurement at one time-point, to a measurement of the same kind at another time-point. In the early days of cancer treatment, the assessments were physical (if the tumor was visible or palpable from the outside), chemical (if some consequence of the tumor’s presence could be measured, for example from body fluid samples), or pathological (if a part of the tumor could be biopsied or otherwise sampled for microscopic examination). Medical imaging has given the physician the ability to observe structures which were not visible from the outside, and to do so minimally invasively. Tomographic imaging has been a powerful development, since patients and tumors are inherently three dimensional. Imaging in three dimensions has provided the ability to delineate the spatial extent of lesions, and has provided the potential for much more accurate measurements.

Imaging modalities provide information about physical properties (e.g. X-ray attenuation, T1 relaxation time) but not biological properties, per se, although the physical properties measured by imaging systems are usually related to biological properties of interest, and may be used to estimate the underlying biological properties quantitatively. In this capacity, different imaging modalities reflect different aspects of tumor growth, for example, x-ray attenuation is highly effective at demonstrating calcifications. Magnetic resonance imaging is very flexible in that it can observe many different aspects of tissue: T1 relaxation time, T2 relaxation time, proton density, diffusion, etc. MR angiography provides the ability to visualize blood flow. MR spectroscopy provides the ability to visualize chemical constituents. Through the injection of a contrast medium such as gadolinium (Gd), regions of breakdown of the blood brain barrier (BBB) may be visualized in a brain MRI, as well as perfusion characteristics of tissue in the brain and other structures. Information related to function rather than structure may also be acquired—functional magnetic resonance imaging (fMRI), for example, provides the ability to image regional brain activation. Positron emission tomography (PET) provides the ability to image function through metabolism. Blood flow velocity and direction may be observed using Doppler ultrasound. Microscopic imaging may be conducted *in vivo* using ultrasound biomicroscopy (UBM) or optical coherence tomography (OCT).

An important problem in medical imaging (and in many other fields) is the explosive growth of data production. Imaging devices are now able to produce a preponderance of data, and computer systems can process and store this information with relative ease. In medical imaging and other fields, the human being remains at the end of the image production pipeline, with the potential to be overwhelmed by the quantity of data being presented. In the context of medical imaging, this has been termed “image overload” (Andriole et al. 2004). To elaborate, in a magnetic resonance imaging study of a patient with a brain tumor, a given patient may be imaged using a variety of pulse sequences: T1, T2, Fluid-attenuation by inversion recovery (FLAIR), T1 with gadolinium (Gd), Proton Density (PD), diffusion, perfusion, MR angiography, magnetic resonance spectroscopy, etc. Further, each sequence is made up of several hundred thousand voxels: historically, the images have been displayed as a series of slices. Advances in data set sizes of CT and MR have shown exponential growth that track advances in computing power (Erickson et al. 2001). The wealth of information produced by efforts to image with progressively finer resolution, with more image contrast types, over progressively larger regions of anatomy should be beneficial to the clinician; it may become problematic, however, if not carefully managed. Important information may be hidden beneath the wealth of data, making looking for the features of interest like looking for the proverbial needle in a haystack. Information may be spread across multiple slices or pulse sequences, making it difficult for the human to assimilate the data, and identify important features (at least when the information is presented as it is today).

In some sense, information overload provides the imperative to use sophisticated computational strategies to provide the radiologist with information more focused on the patient and the clinical task (e.g. biology and disease related information) rather than physics centric information which has been presented historically. Plain-film x-ray imaging systems have provided radiologists with x-ray attenuation information, because that was what film could provide. In the past, in the limited cases in which post-acquisition image processing systems have been applied, these have been relatively simple. Hounsfield units have in some cases been thresholded in CT to define tissue types and then used to present colored 3D images, and Doppler ultrasound images have often been thresholded to help identify areas of flow above a certain range. However, because of an historical lack of computing algorithms, and because of limits to the types of information and numbers of measurements provided by imaging systems, it has been difficult for imaging systems to make many sure judgments, beyond these relatively simple examples. Minimally processed physical information was what was presented to the radiologist, because that was what was sure, and this has continued into current medical practice—radiologists still examine T2 weighted images, when what they are really interested in are the biological properties: tumor growth, inflammation, infarction, etc. Likewise, physicians have historically examined a selection of volumes at multiple time-points, when what they are really interested in is what has changed. However, there may now be a shift in the form of presentation. The explosion of data produced by CT and MR scanners has made it both necessary and possible for the radiologist to be presented with processed data which is more reflective of the underlying biological properties, which are what is of interest, instead of the measured physical properties (which the radiologist was likely going to use to infer the biological properties of interest). If the radiologist is interested in change in a known process compared to a prior time point, then the radiologist should be given an image of change, and these changes should reflect the underlying biology to the greatest degree possible.

The benefits of and problems associated with rapidly increasing quantity of data acquisition is by no means specific to medical imaging, but instead is occurring across a gamut of disciplines ([NoAuthorGiven], 2006). Likewise, the desire to identify changes between serial images and efforts to use computers to help accomplish this task has not only been described within the context of medical imaging (Patriarche and Erickson, 2004), but is also being simultaneously explored in other imaging fields including remote sensing, astronomy, surveillance, geology, and others (Radke et al. 2005; Bovolo and Bruzzone, 2005; Lu et al. 2004; Coppin et al. 2004). There is additionally extensive work being undertaken in the neuroscience of change perception (Simons and Levin, 1997; Rensink, 2002; Thornton and Fernandez-Duque, 2002; Simons and Rensink, 2005; Noë et al. 2000; Levin et al. 2000) (i.e. how people compare images in their minds when they do so manually). It is very likely that researchers involved in the development of change detection techniques could benefit from familiarity with this growing body of literature, because understanding the neurologic mechanisms behind successful manual identification of changes, and understanding the situations in which and reasons why clinicians fail to recognize changes may suggest computational strategies for automated support.

## Change Detection and Characterization

Establishing the presence or absence of change over time, and characterizing those changes, is a common clinical task and a key motivation for the acquisition of serial imaging studies—the goals of this task contrast with those of acquiring single time-point imaging studies in order to explain symptoms and diagnose disease. In current clinical practice, the former is in some respects more challenging than the latter, first because serial analysis requires the examination of at least twice the quantity of information compared with single time point analysis, and second because side-by-side comparison requires the radiologist to perform a mental comparison function after assessing each time point, based upon memory of both acquisitions. Making the task even more challenging is the fact that data produced by current state of the art scanners is confounded with a variety of acquisition-related factors (e.g. signal nonuniformity and noise, patient positioning inconsistencies, modality-specific artifacts, etc.), and these factors may change from the time of one acquisition to the next, so in order to generate a mental map of the changes, the clinician must mentally filter out these confounds. A selection of methods, mathematical formulations, and computer algorithms have been used to compare serial volumetric images—to identify, and in some cases localize, changes (Patriarche and Erickson, 2004). These have utilized greater and lesser degrees of automation, and have shown greater and lesser degrees of effectiveness.

### “Manual” approaches

Methods focused on reducing the image data to concise quantitative measures are more relevant in the context of research, where it may be desired to compare the response of one group of patients (enrolled in the ‘control’ arm) with another group of patients (enrolled in the experimental treatment arm). Various approaches have been taken in the past to produce such simple metrics. Several groups have used maximal diameter and related methods. In one such method, response evaluation criteria in solid tumors (RECIST) (Padhani and Ollivier, 2001; Gehan and Tefft, 2000; Therasse et al. 2000; Tsuchida and Therasse, 2001; Padhani and Husband, 2000), lesions larger than 1 cm are identified, the largest in-plane diameter of each is measured, and an overall sum is computed. Two related methods, the World Health Organization (WHO) method (Miller et al. 1981) and the Southwest Oncology Group method (Green and Weiss, 1992), use largest diameter and largest corresponding perpendicular. In these methods, “largest diameters” are used to capture the extent of the tumor. Other groups have used volumetrics, in which the boundary of the tumor or other region of interest is defined, for example by outlining, and then the total volume of abnormal appearing tissue is summed (Rusinek et al. 1991; Weiner et al. 2000; Jack et al. 1998; Haney et al. 2001a; Haney et al. 2001b).

Single-diameter methods are very effective at reducing data. The measures produced are intended to reflect patient status, or when computed for two serial acquisitions and then compared, of response to therapy or progression of disease. In spite of this, there are two chief problems with these methods: one is that they do not adequately capture the status of the patient (Patriarche and Erickson, 2004), and the other is that they suffer from a high inter- and intra-rater variability (Thiesse et al. 1997; Filipek et al. 1991; Clarke et al. 1998). In some respects, the tasks these methods pose to the clinician are poorly constructed: RECIST for example, assumes a reliably definable diameter, but many tumors are infiltrative, and thus may not present a discrete boundary. By focusing on maximal in-plane diameter, they implicitly assume that tumors grow uniformly in all directions, but this is certainly not the case. Changes in image acquisition parameters or imaging plane can cause changes in the measurement even though the tumor is actually unchanged. In some respects, such methods are also poorly matched to the human ability to perceive: the clinician cannot necessarily easily tell by visual inspection which is the largest diameter, and thus their measures are not deterministic and do not always reflect the maximal extent of the tumor. The methods do not always measure all features important to the assessment of changes in status: in addition to changing in extent, tumors may also change in character (for example, in terms of internal signal properties or margin appearance). Rate of change may also differ greatly from one location within the tumor to another, and thus it may be very important to consider the inherent heterogeneity of tumor cells.

### “Automated” approaches

“Manual” approaches are to a great degree directed towards simplifying the problem, requiring interaction with as little data as possible at a time, and taking as little time as possible to perform. Computational approaches may be quite complicated in comparison (but are still intended to be relatively simple from the perspective of the user), and may produce important performance improvement from the perspective of the radiologist. Even anatomic alignment can improve the performance of radiologists [Erickson CI, 2006]—and this one step greatly facilitates further computational steps, such as subtraction. Computers may be programmed to perform arbitrarily complicated tasks perfectly deterministically (or, if desired, maximally deterministically, which can sometimes be advantageous), often showing better performance as the volume of data increases. This is important, because this means analysis strategies do not have to be performed upon drastically simplified versions of the data— the analysis processes can be quite complicated, and can produce extremely rich and tailored output. Although some express skepticism that such systems can work in practice, some authors have shown it to be possible to construct these systems (Patriarche and Erickson, 2006a), and to make them extremely automated and produce highly rich information as their output. These systems have further been shown to offer enormous potential value to clinicians (Patriarche and Erickson, 2006b).

Some approaches to computer-assisted change detection focus on localization of changes, whereas others focus on reduction of the data at the output stage. A relatively simple and intuitive computational approach which has been used for change detection and localization is registration followed by subtraction; this method has been demonstrated to offer a high degree of sensitivity to changes (Hajnal et al. 1995a; Hajnal et al. 1995b). Some authors have used non-linear registration/warping, and in some cases, such as when the tissue is deformable and such deformation is what is of interest, these approaches are very natural and appropriate (one example would be when it is of interest to determine whether the ventricles of the brain are being compressed by mass effect). Other approaches have combined the ability of approaches such as subtraction to produce localized descriptions of change, with the ability of approaches such as diameter-based approaches, volumetrics, and other measurement sampling approaches to produce concise quantitative summaries of change (Patriarche and Erickson, 2006a, Patriarche and Erickson, 2006b).

## Benefits of the Direct Computation of Change

**Figure 1:**
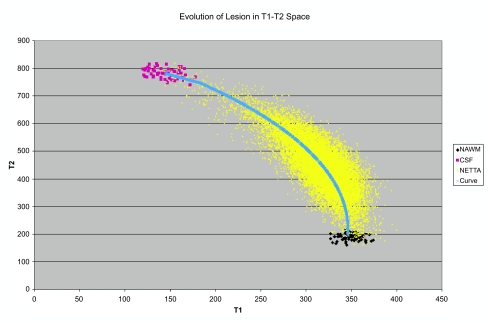
Scatterplot in T1–T2 space showing samples of normal-appearing white matter (NAWM, navy blue), cerebrospinal fluid (CSF, magenta), and non-enhancing T2 abnormality (NETTA, yellow) for a brain cancer patient. As normal appearing white matter acquires greater abnormal character, its T2 intensity increases and its T1 intensity decreases. A trajectory is followed through feature space, and from the perspective of quantifying lesion character, variation in the direction of this trajectory is what is most important. Specifically, a voxel half-way along this line between the NAWM centroid, and the CSF centroid, might be said to be 50% abnormal, while a voxel three-quarters of the way along this line might be said to be 75% abnormal. By focusing on fractional shifts in the position of voxels in feature space along this trajectory, very subtle changes in character may be detected. At the same time, variation in the position of a voxel perpendicular to this line may be treated as being due to noise. In the above figure, the blue line labeled ‘curve’ has been fitted by a computer program in order to emphasize the trajectory.

The emphasis on change assessment, rather than size assessment, is purposeful. The direct computation of change from the total body of data (as opposed to computation of some measure from the data from each time-point and subsequent comparison of the measures from the two time-points) can help to overcome a variety of problems. Two acquisitions provide more information than one, and this incremental information might be used to help resolve ambiguity. The acquisition from one time-point can provide a standard of reference for the other. Artifacts, for example, are relatively unlikely to present identically in multiple acquisitions. By applying a dedicated change detection method which uses more than one acquisition, these problems may be reduced. A dedicated change detection algorithm may implicitly manage artifacts, using the fact that changes resulting from artifacts being present at one time-point but absent or different at the other are relatively unlikely to manifest in the same way as real changes, which typically manifest in particular ways (Figure 1). An algorithm might also explicitly manage artifacts, for example by using knowledge of specific varieties of artifacts (e.g. RF inhomogeneities)—by examining and comparing regions of normal tissues at both time-points, and by developing a mathematical description of the artifact in the particular case for use in intermediate stages of processing. Using such a method, the impact of RF inhomogeneities at both time-points might be reduced not only in regions of normal tissue, but through inference and models, in regions of pathology (including changing pathology) as well.

Boundaries of interest may be defined by the extent of the changing region, rather than by the extent of the abnormal appearing region (this is in contrast with the approach taken by size methods such as volumetrics). The delineation of tumor boundary, whether by manual or automated means, will always have error. If the actual change encompasses only a fraction of the entire lesion, then the cumulative error incurred as a result of delineating the boundary of the entire lesion twice (once at each time-point) could easily overwhelm the change measurement (and it would not describe changes in character). Attempting to identify the regions of change, directly, rather than defining lesion boundaries at each time point, avoids this source of error.

In the context of therapy response assessment, the nature of the change may be of greater interest than its absolute size. Imaging based measurements frequently do not reflect total lesion burden (Kelly et al. 1987a; Kelly et al. 1987b; Burger et al. 1988; Johnson et al. 1987; Tovi, 1993; Tovi et al. 1994), but changes in the imaging appearance are very likely to reflect changes in the underlying disease.

A subtle lesion seen in imaging could also be ambiguous, and may or may not be related to the disease under investigation. Change computed from serial imaging studies may be a relatively simple way to help disambiguate these questionable features, as *change* may provide greater sensitivity and specificity than static intensity characteristics, or imaging appearance.

### The ability to detect changes of subtle degree

The ability to detect *subtle* changes, in both structures which were previously normal and in structures which were previously abnormal, is an area of great potential for computer methods. The ability to detect subtle changes allows earlier evaluation of treatment effects and thus, earlier intervention if a treatment is failing. Changes due to a therapy may also be transient, and may be quickly overwhelmed by progression, but these changes may still be of interest to the researcher, and it is necessary to have a mechanism to measure these changes. Mechanisms to detect subtle response from short-interval acquired scans may help identify which patients are responders and which are not, at least as effectively as genetic profiles. Manual detection of these subtle changes is very challenging; change detection algorithms may identify and characterize predictive changes before they are obvious and definite with the unaided eye.

### The ability to detect changes of small size

Related to the issue of the detection of subtle changes is the ability to detect spatially small changes—the difference being that subtle changes are changes which are subtle in character but which may (or may not) encompass a large spatial extent; whereas small changes are changes covering a small spatial extent but which may or may not be subtle in character. Visually comparing images in a ‘side-by-side’ mode cannot be as sensitive to small changes as computer algorithms that can compare ‘in-place’. Visualization modes that allow ‘in-place’ comparison (such as ‘flicker display’) can increase sensitivity to small changes, but computer algorithms are still likely to be more effective.

### The ability to localize changes

Methods of change detection which produce localizable measures of change are likely to be very useful. To the clinician, the location of progression may be important since it may help to determine which symptoms can be attributed to disease progression. For example, in melanoma lesions observed using ultrasound biomicroscopy or optical coherence tomography, a change indicating penetration of the basal cell layer versus superficial layers has significant implications for treatment and prognosis. In brain tumors observed using MR or CT, whether or not the tumor is infiltrating critical structures such as eloquent cortex likewise has great implications for the patient and for the future behavior of the disease. Localizable descriptions of change may also provide information regarding which regions of the tumor are more aggressive. This may be important from a clinical stand-point, if the clinician wishes to biopsy the most aggressive parts of the tumor. It may be important from a research stand-point, because the researcher might wish to biopsy both the more aggressive regions of the tumor, and separately the less aggressive regions of the tumor, and perform comparative genomic analysis, to help to better understand what makes the category of tumor aggressive or not. In the context of individualized medicine, the inherent heterogeneity of tumors is very important because the intent in individualized medicine is to tailor the therapy not just to the patient’s genome, but perhaps more importantly to the disease as well. Imaging-based change detection may offer the best possibility for optimal therapy, by enabling targeted biopsy and additionally by providing a description of the response to therapy of all regions of the tumor.

## Considerations Guiding the Development of a Change Detection System

### Practical considerations

Seven criteria may be proposed to guide the development of change detection and characterization methods. 1) The measures minimize the effect of non-biological changes (for example acquisition related issues). There are a variety of ways this objective might be approached, including attempting to explicitly reverse the effects of the confounds or the method might be made to be insensitive to the confounds. 2) Minimize inter- and intra-observer variability. In the context of the clinic, reduced variability means greater confidence in diagnoses, fewer mistakes, and a greater willingness to act earlier. In the context of research, reduced variability means that smaller cohorts and/or shorter time periods are required to achieve desired power. 3) The measures should require as little human effort as possible. 4) The computed measures should correlate with or predict the clinical status of the patient. 5) The measures should provide localizable descriptions of change. 6) The final measures should be intuitive. 7) The measures should be quantitative (or some aspects of them should at least be expressible quantitatively), so that they may be compared using established statistical approaches, and summarized in research articles or clinical documents.

### Technical considerations

There are a number of key technical points which should be considered during the development of these algorithms.

1. It is feasible to suppress noise, artifact, and other confounding factors by using knowledge about imaging and biology. Knowledge about tissue properties, and imaging characteristics may be incorporated into change detection and characterization algorithms.
2. Change, like discrete anatomical structures, tends to exhibit coherence in space, in contrast with noise which usually does not. Spatially contiguous regions which exhibit similar traits (e.g. all changing in the same way) may therefore be detected with greater sensitivity than a pixel-by-pixel method could achieve.
3. Changes may be identified in terms of character as well as extent (although the latter may be identified because it manifests as one sub-type of the former). This separation of tasks is important for a variety of reasons, particularly for multi-parametric images. There are times when the extent of the lesion is unchanged, but the character does change. It is also possible to have opposing findings (one image type indicates decrease, while another indicates increase) within a complete examination. By computing a character change image, one may identify these more complex changes, compared with a simple computation of extent. A classic example of this is that on most MRI pulse sequences, a small amount of edema in white matter signals like gray matter. An algorithm focused on boundaries might miss this finding, and assign substantial changes to white matter and gray matter volumes. An algorithm that looked at the nature of change (e.g. this was white matter, therefore, what now signals like either gray matter or white matter with some edema must be the latter) will be more accurate than one focused strictly on either extent or character.

### The role of artificial intelligence and a priori knowledge

Change detection and characterization has been proposed as a new field of computer-aided diagnosis (Khorasani et al. 2006). As in other fields of computer aided diagnosis, change detection and characterization algorithms will be built upon a foundation of image processing *and* knowledge. The example above in which white matter with minimal edema ‘looks like’ gray matter is a good example of the importance of knowledge. Humans are able to distinguish this confounder because of knowledge about where white matter and gray matter are expected to be. In addition, there is knowledge about MRI, indicating that white matter with slight edema does appear like gray matter.

### Essential steps in change detection and characterization systems

From an algorithmic stand-point, there appear to be a number of essential elements of change detection and characterization systems. The ideal formulation has not yet been determined, although initial studies have been done. Our own current change detection system implementation is architected as a processing pipeline and so we will describe these elements as steps.

The first step in successful change detection is suppression of acquisition-related confounders. Obviously, one should avoid intentional change, and work to minimize other sources of variation in acquisition. In the case of serial MR examinations, possible confounds include changes in patient position, RF heterogeneity, intravenous contrast changes (time of contrast administration, contrast type, contrast concentration), acquisition parameters, etc. It is usually necessary to perform pre-processing to attempt to remove at least some of these acquisition related changes.

In the case of MRI, it is necessary to manage the effects of changing acquisition parameters/contrast characteristics that occur in clinical practice. The quantitative values of the intensities of different tissues may vary dramatically from one acquisition to another, due to changes in acquisition parameters and other factors. To address this problem, we have developed an algorithm that uses knowledge about anatomy and MR sequence properties to generate reference tissue samples in patient images (Patriarche, 2004). Despite the fact that the specific intensities may change from one acquisition to another, there are specifics about the intensities which don’t change. For example, the algorithm can make use of the knowledge that the fractional representation of tissues in a partial-volumed voxel (e.g. white matter on the edge of a CSF-filled cavity) will manifest in feature space as a linear combination of the multispectral intensities of the contributing tissues, with the weighting of the contribution of the multispectral intensities of the contributing tissues, being equal to the fractional volumetric representation of the contributing tissues. To elaborate, if the centroids of the tissues represented in a particular voxel are known, the algorithm may mathematically generate a line connecting the centroids of the contributing tissues in multispectral intensity space. Then, the position of a particular voxel along that line indicates the fractional volume contribution of the contributing tissues. Because the effects of changing contrast properties are never explicitly reversed, this is an example of managing acquisition related effects without attempting to reverse them—managing the issue (in this case contrast property changes) by using an algorithmic approach unaffected by the issue.

The next step in change detection involves categorization of the contents of voxels, into the tissue(s) they appear to contain (e.g. white matter, gray matter, or CSF in the brain) based on image intensities and using knowledge of how tissues appear, in conjunction with a priori anatomical information. It is important to note that our system explicitly focuses on the space between ‘clusters’ in feature space, instead of on the clusters themselves—i.e. the algorithm is specifically focused on identifying and characterizing tissue mixtures within a voxel. This is a critical part of what facilitates the algorithm’s ability to detect subtle changes. One effect of the assignment of voxels to transition categories is noise reduction, because these transition categories are constrained in their possible multispectral intensity characteristics, and variations from these known multispectral instensity characteristics may thus be attributed to noise and disregarded.

The next step is to detect changes in the character of tissue and to assign changes in the extent of each tissue type. A growing tumor will result in a change in extent of tumor, but also a change in the boundaries for the adjacent normal tissues (due to mass effect); these deformations could be measured with non-linear registration algorithms. An example of change in character is increased T2 signal within a structure; these could be measured with algorithms specifically designed to measure this kind of change (Patriarche and Erickson, 2006a). In some portions of the image which would be expected to exhibit large discrete boundaries, finite element model representations of boundaries might be appropriate, which would be a means to achieve sub-voxel resolution, allowing the detection of sub-voxel shifts in boundary position. Development of the finite element model would require the application of extensive a priori knowledge of anatomy and imaging characteristics. For deformable tissues, non-linear registration/warping could be performed at this point, under the constraints of the finite element model and knowledge of the ways that tissues can and can not change from one time point to the next. Obviously warping algorithms would have to be applied under the knowledge that tissues will almost definitely not retain their imaging intensity characteristics (i.e. changes in character are expected to occur—as mentioned above), but in locations where anatomy will be retained (though possibly warped) from one acquisition to the next, intensity characteristics will remain consistent with the type of tissue contained in the region at both time-points. Warping, in the language of mathematics, is an ill-posed problem, at least from the perspective of the imaging data. Of course, there is a correct solution, but there is not a unique solution considering only the imaging data. An essentially unlimited number of warping fields might be developed to explain any deformations which might exist. Additionally, an essentially unlimited number of trade-offs between changes in character and changes due to deformation may be developed.

Warping algorithms offer an opportunity to detect changes which do not themselves directly cause intensity changes—at least not at the site of interest (i.e. at the site of tumor deposition). Specifically, multivariate calculus operations may be performed upon the derived warping field to help identify sites of tumor deposition which may or may not directly exhibit abnormal imaging intensity characteristics (Thirion and Calmon, 1999), which should be considered to be important since one consequence of the nonequivalence between what imaging systems measure (physical properties) and what clinicians seek (information regarding pathology) is that tumor does not always appear in imaging to be distinct from its surroundings (Kelly et al. 1987a; Kelly et al. 1987b; Burger et al. 1988; Johnson et al. 1987; Tovi, 1993; Tovi et al. 1994), but more likely it may be expected to displace its surroundings. A warping step would additionally help to simultaneously establish true and correct correspondence between voxels from one acquisition to the next.

After the changes have been quantified, it is likely that most voxels will possess measurable changes even if there is no underlying biological change, strictly due to noise. Such noise may be identified and eliminated by tests of spatial coherence, because changes due to underlying biological processes tend to exhibit spatial coherence, whereas changes due to noise do not. The algorithm may determine whether spatially contiguous regions are changing in the same manner (i.e. an entire spatially contiguous region of the patient’s anatomy which is acquiring T2 abnormality). Regions which are too small in extent, or which are too subtle to be certain that they are real, may then be discarded.

## Validation

### Technical validation

As in many technology validation problems, developers of change detection algorithms must document that their algorithms are detecting and characterizing an imaging change correctly. Additionally, it is important to document that the changes are due to biologically important changes (e.g. tumor progression). Survival of the patient has often been used as the ultimate standard, but is now falling out of favor because unrelated factors can affect survival, and survival increases the time and cost of performing clinical trials.

There are several ways that one might validate change detection methods from a technical perspective. Creating a family of phantom images with known changes is one commonly applied technique. Such a phantom could be very simple—phantom images may not appear very similar to real images. However, such simple phantoms are ideal for evaluating specific components of change detection algorithms. One might create a family of phantoms to investigate such aspects as the effect of background and noise, performance on low contrast/diffuse changes, higher contrast but physically small changes, changes in shape versus changes in intensity, effects of adding more than 2 time points, adding more signal channels, and so on. It is possible to create phantoms that very closely model the imaging device properties, even if the objects in the image do not appear much like biologic objects. This can allow one to create a single phantom with a range of changes that can more easily demonstrate strengths and weaknesses of algorithms than morphologically correct phantoms (Patriarche and Erickson, 2006a). Once each component has been optimized, they are combined to create a change detection system. Evaluating that system will require either a more complex phantom, or it may then be applied to biologic data.

For cases where morphology is important, there are some resources already available which can produce morphologically correct phantoms which can also simulate real imaging devices, including the ability to add noise or some basic artifacts like field shading in MRI (Cocosco et al. 2006; Cocosco et al. 1997; Kwan et al. 1999; Kwan et al. 1996; Collins et al. 1998).

One must also consider how to assess phantom or real image changes. One may wish a global binary response: *is there any* change between a pair of images. Another type of response would be to count *how many* pixels are changing. Finally, one could ask for a relative scale of *how much* change is occurring in each pixel. For each of these types of change measures, one could then identify true positives, true negatives, false positives and false negatives. This would then allow computation of standard metrics of performance such as sensitivity, specificity, accuracy, as well as measures more frequently used for classifiers such as Percent Correctly Classified, the Jaccard Coefficient, or the Yule Coefficient.

### Clinical validation

A criticism of most phantom methods is that they do not fully represent the properties of the images seen in clinical practice. For this reason, it is necessary to validate change detection algorithms using real patient/subject data. The challenge is that getting ground truth may be difficult with living subjects. Although it is quite possible to get multiple examinations on a patient or an animal, it is difficult to get ‘ground truth’ for consecutive imaging time points. Getting that ground truth through invasive means (biopsy at the time of an examination) would likely confound subsequent imaging examinations. Traditional biopsy methods require removing tissue to see what is there. One must then assume that what is left behind is the same—an assumption that is not always correct. Furthermore, the procedure of collecting the tissue may induce changes (e.g. hemorrhage, scarring) that are not the changes of interest, but which will be changes to the algorithm. One could get reasonable ground truth for the last time point if the tissue is removed *en bloc* and correctly aligned with imaging. Animal models in particular may allow collection and fixation of the entire brain, allowing the best chance for good alignment of tissue specimens with imaging.

A common strategy for evaluating imaging methods is to use multiple human experts to develop a panel consensus about the image(s) that should represent truth. The weakness in this case is the assumption that the change is of a size and magnitude that it will be detected by most human experts. We have shown that using the computer to re-align images will help observers more accurately detect changes, and this may help define a better ground truth (Erickson, Submitted). If they are aligned, the so-called ‘flicker’ display method has been shown to be useful for helping humans to detect subtle changes between 2 images. But neither of these methods helps humans to detect changes that span multiple image types. Therefore, while the panel consensus model may be helpful for some aspects of validation (e.g. “does change detection reduce the variability of observers?”) it may not be helpful in documenting that it improves sensitivity.

An alternative strategy for validating algorithms using real images is based on the premise that consistency is nearly as valuable as accuracy, as long as one is reasonably sensitive. In this case, one might collect 3 serial examinations on a subject, which we will refer to as A, B, and C. For most types of output, one should expect that the change from A to B plus the change from B to C should equal the change from A to C. Of course, it is important to define the transform functions: if one is simply producing a binary output like progression or no progression, then the math would be 1+1=1 where 1 = progression and 0 = no progression. The same math might apply if one considered the images on a pixel by pixel basis with each pixel being labeled as progression (1) or no progression(0).

For cases where the output is not binary, one might consider summing the changes in a given category (e.g. the net volume change of a certain type for A to B plus that change for B to C should each the net change of that type for A to C). It should also be reflexive—that is, A to B should equal the inverse of B to A. For this general class of methods, it is essential that a reasonable accuracy be demonstrated—an algorithm that arbitrarily set every pixel to showing change/progression would meet the above criteria but not be very useful.

Another way one might document the clinical value of a change detection system is if one could demonstrate that it correctly predicts future (humanly detectable) progression based on early, short-interval scans. In this case, one might obtain a baseline examination, plus an examination early in the course of treatment and use the change detection algorithm to identify signatures that predict that the tumor will visibly respond (or not). If one could determine that a tumor is not responding early, one could switch to an alternative therapy early. This might improve patient survival while also reducing costs by avoiding continuing an ineffective therapy.

There are other evaluations that are of interest, including documenting the impact of change detection on the human faced with image overload; one could evaluate the impact on efficiency. While this is an important socio-economic problem, it is not directly germane to understanding cancer, and will not be described further.

## Conclusions

Change detection and characterization is a burgeoning field, located at the confluence of: rapidly increasing quantity of image data acquisition; the development of new therapies and the need to objectively evaluate them; and continued increase in the availability of inexpensive computational power. It is expected that this field will continue to develop, and that change detection and characterization will both benefit from and alleviate the problems associated with image overload, and will help clinicians to fully utilize the information at their disposal. It is expected that the ability of change detection and characterization systems to generate quantitative and reproducible measures will facilitate the development of objective definitions of disease progression and regression, and these in turn will help to select the most effective therapies. Methods for evaluation of change detection and characterization technologies will continue to be an important facet of this rapidly progressing technology.
